# Development of posttraumatic stress disorder symptoms after intensive care - how to prevent it?

**DOI:** 10.1192/j.eurpsy.2022.216

**Published:** 2022-09-01

**Authors:** T. Teixeira, J. Quarenta, S. Martins, A. Almeida, B. Ribeiro

**Affiliations:** Centro Hospitalar do Tâmega e Sousa, Departamento De Psiquiatria E Saúde Mental, Penafiel, Portugal

**Keywords:** delusional memories, intensive care unit, post-traumatic stress

## Abstract

**Introduction:**

Over the last decade, there has been identified that critical illness survivors have high rates of psychiatric disorders such as posttraumatic stress disorder (PTSD). The experience of admission to intensive care units (ICU) and illusory memories may cause short and long-term psychological disorders.

**Objectives:**

To evaluate psychiatric disorders, such as PTSD, after ICU discharge, and determine the prevalence, risk factors, and prevention strategies for PTSD in these patients.

**Methods:**

Non-systematic review through research in PubMed. Addicionally, a case report will be exposed, after the patient was diagnosed with SARS‑CoV‑2 and stayed in ICU for more than 30 days.

**Results:**

The development of PTSD has been related to the number of adverse memories patients recall from their ICU experience. Some studies have shown that approximately 47% of patients remember real facts and 34% have illusory memories relative to their stays in the ICU. There were identified some risk factor associated to the increased risk of post-ICU PTSD, such as early post-ICU memories or psychotic experiences, pre-ICU psychopathology, benzodiazepine sedation during ICU and substantial acute stress symptoms occurring < 1 month after exposure to a traumatic stressor.

**Conclusions:**

High levels of anxiety and the development by patients of PTSD are being recognized as significant problems occurring after a stay in an ICU. The results of this study highlight the need to recognise the risk factors and to establish a early follow-up after ICU stay. This way is possible to identify patients who are at risk of developing acute PTSD-related symptoms, and early intervention can be institued.

**Disclosure:**

No significant relationships.

